# Using eDNA metabarcoding to understand the effect of fire on the diet of small mammals in a woodland ecosystem

**DOI:** 10.1002/ece3.9457

**Published:** 2022-11-08

**Authors:** Saumya Wanniarachchi, Matthew Swan, Paul Nevil, Alan York

**Affiliations:** ^1^ School of Ecosystem and Forest Sciences The University of Melbourne Creswick Victoria Australia; ^2^ Trace and Environmental DNA Laboratory, School of Life and Molecular Sciences Curtin University Perth Western Australia Australia

**Keywords:** environmental DNA, fire regime, pyrodiversity, resource selection, scat

## Abstract

Food acquisition is a fundamental process that drives animal distribution and abundance, influencing how species respond to changing environments. Disturbances such as fire create significant shifts in available dietary resources, yet, for many species, we lack basic information about what they eat, let alone how they respond to a changing resource base. In order to create effective management strategies, faunal conservation in flammable landscapes requires a greater understanding of what animals eat and how this change following a fire. What animals eat in postfire environments has received little attention due to the time‐consuming methodologies and low‐resolution identification of food taxa. Recently, molecular techniques have been developed to identify food DNA in scats, making it possible to identify animal diets with enhanced resolution. The primary aim of this study was to utilize eDNA metabarcoding to obtain an improved understanding of the diet of three native Australian small mammal species: yellow‐footed antechinus (*Antechinus flavipes*), heath mouse (*Pseudomys shortridgei*), and bush rat (*Rattus fuscipes*). Specifically, we sought to understand the difference in the overall diet of the three species and how diet changed over time after fire. Yellow‐footed antechinus diets mostly consisted of moths, and plants belonging to myrtles and legume families while bush rats consumed legumes, myrtles, rushes, and beetles. Heath mouse diet was dominated by rushes. All three species shifted their diets over time after fire, with most pronounced shifts in the bush rats and least for heath mice. Identifying critical food resources for native animals will allow conservation managers to consider the effect of fire management actions on these resources and help conserve the species that use them.

## Graphical abstract

Information regarding diet changes over time after disturbance is lacking for many animals living in habitats prone to disturbances such as fires. DNA metabarcoding is a versatile molecular method that allows simultaneous characterization of many biotas using taxonomically informative barcode regions. In this paper, we utilized eDNA metabarcoding to obtain an improved understanding of the diets of three native Australian small mammal species: our results suggest that all three species shifted their diets over time after fire.

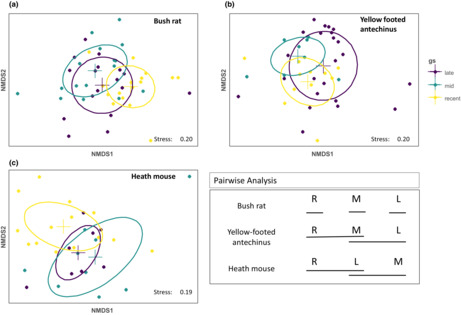

## 1 INTRODUCTION

Understanding how animals utilize resources in different ecosystems is essential to ensure effective conservation practices. Food is a direct resource utilized by an animal in real‐time, making diet a robust indicator of resource usage; furthermore, food availability and selection directly influence the survival and persistence of animals which subsequently will be reflected in patterns of co‐occurrence (Broughton & Dickman, 1991; Di Stefano et al., 2014; Fischer et al., 2006; Whittaker et al., 1973). Variations in diet will depend on food availability, species dietary preferences, and life‐history strategies (Ozaki et al., 2018). Thus, accurate information on diet helps identify how species interact with their environment and persist under changing conditions (Clare, 2014; Monterroso et al., 2018).

Fire is a disturbance that can change food availability in time and space by altering nutrient cycles, community assemblage, and habitat structure (Bowman et al., 2016; Gill, 1975). Fires consume above‐ground vegetation, but in fire‐adapted ecosystems, most organisms survive or recolonize when resource requirements are met, responding to different successional stages as habitat suitability changes (Brown et al., 2013; Whelan, 1995). In many fire‐prone environments, fire is used as a land management tool to promote biodiversity and to lower wildfire risk (Gill, 1975; Whitehead et al., 2003). Several studies have indicated the importance of understanding the influence of fire on the food resources of animals as fire can alter or limit critical food reserves (Lashley et al., 2015; Stojanovic et al., 2020; Valentine et al., 2014). However, insufficient knowledge on what food resources animals consume in postfire environments limits the effective usage of fire for faunal conservation in flammable landscapes (Driscoll et al., 2010; Kelly et al., 2017). Notably, as managers strive to maintain a diversity of postfire habitats across the landscape (“pyrodiversity”), we need to better understand how resource use changes over time.

What animals eat in postfire environments has been given little attention compared with studies of their responses to other resources such as vegetation structure and shelter (Anderson et al., 2018; Dawson et al., 2007; Di Stefano et al., 2014; Geary et al., 2020; Southgate & Carthew, 2006). This lack of information could be attributed to the time‐consuming identification of food items or ethical concerns related to methodologies (Klare et al., 2011). Traditional methods of assessing diets include direct observations, analysis of prey remains, stomach content, or scat (Stoddart, 1979). Scat analysis is an extensively used, noninvasive method to assess animal diet (Dickman & Huang, 1988). For animals caught in traps, food items present in scat are likely to express foraging events close to an animal's capture time, making scat analysis an effective method to disclose information on diets (Di Stefano et al., 2014; Dickman & Huang, 1988). A well‐established method of analysing scat involves macro and micro‐histological identification (Storr, 1961); however, physical digestion through mastication and chemical digestion in the gut can result in diet items that are difficult to differentiate using histological methods. Thus, histological samples cannot be identified to a finer taxonomic resolution in many cases (Klare et al., 2011; Storr, 1961; Zeale et al., 2011). Furthermore, soft tissue and liquid food items (e.g., nectar) can remain unnoticed in histological analysis (Taberlet et al., 2018). Methods such as stable isotope identification, macromolecule analysis, and DNA‐based identifications can address this disparity (Nielsen et al., 2017). Animal scat is a combination of different diet items or, more clearly, a partially digested mixture of DNA from different food species; thus, investigating the DNA in scat samples could result in a higher resolution of taxonomic information than histological methods.

Modern molecular tools have facilitated the analysis of mixed samples, typically from the environment, for the identification of species present via their DNA. Trace amounts of DNA isolated and characterized from biological substrates including scats, soil, water, or air, are collectively referred to as environmental DNA (eDNA; Taberlet, Coissac, Hajibabaei, & Rieseberg, 2012). When combined with Next Generation Sequencing (NGS) technologies, eDNA metabarcoding (Taberlet, Coissac, Pompanon, et al., 2012) can provide information on, for example, community composition, food web dynamics, animal diet, and invasive or pest species presence/absence (Ruppert et al., 2019; Taberlet, Coissac, Pompanon, et al., 2012). Thus, eDNA metabarcoding is an ideal method to identify what animals eat in postfire environments with high resolution and precision. From the handful of studies investigating animal diets postfire, metabarcoding work is rare (Anderson et al., 2018). Nevertheless, metabarcoding technology is rapidly developing with a wide range of molecular databases and can be used to better understand the role of fire in determining resource use.

The aim of this study is to utilize eDNA metabarcoding to obtain an improved understanding of the food resources and effects of fire on the diets of three native Australian small mammal species: yellow‐footed antechinus (*Antechinus flavipes*), heath mouse (*Pseudomys shortridgei*), and bush rat (*Rattus fuscipes*). Previous work on the diets of these species is limited, but in general, yellow‐footed antechinus diet predominantly consists of invertebrates gleaned from ground litter, logs, tree trunks, and stumps (Hindmarsh & Majer, 1977; Kelly, 2006; Lada, Nally, & Taylor, 2008). Individuals are also reported to consume nectar from the flowers of a range of shrubs and trees (Menkhorst et al., 1995). The heath mouse is a generalist herbivore, feeding predominantly on plant stems, flowers, and seeds, although some insect and fungal material are also consumed (Braithwaite et al., 1978; Watts, 1977). The bush rat is an opportunistic omnivore, eating arthropods, seeds, fruits and other plant tissue stems and leaves, and fungi (Carron et al., 1990). The diets of these species vary seasonally (Carron et al., 1990; Cheal, 1987; Di Stefano et al., 2014); however, little is known about how disturbance such as fire affects their diets. Here we use an emerging methodology, eDNA metabarcoding, to determine: (i) How do the overall diets of the three small mammal species differ? (ii) Does diet change over time after fire? An improved understanding of resource requirements will assist land managers to better conserve these species at a landscape scale.

## 2 METHODOLOGY

### 2.1 Study area and site selection

The study area is located in south‐western Victoria, Australia, across ~150,000 ha. It lies within a roughly rectangular region marked by the towns of Edenhope (37°02′24″S, 141°17′20″E) and Dartmoor (37°55′41″S, 141°16′21″E) in Victoria and Naracoorte (36°57′21″S, 140°44′23″E) and Mount Gambier (37°49′23″S, 140°46′54″E) in South Australia. The area includes parks and reserves containing native vegetation, extensive tracts of pasture, and privately managed eucalypt and pine plantations. This study forms a component of a larger project studying the responses of animals to fire in a fragmented landscape (Delaney et al., 2021; Nalliah et al., 2021). In parks and reserves, the predominant vegetation type is open heathy woodland where the canopy is dominated by *Eucalyptus* species such as Brown Stringybark (*Eucalyptus baxteri*) and Desert Stringybark (*E. arenacea*), with a sparse understory dominated by Grass Trees (*Xanthorrhoea australis* and *X. caespitosa*), *Acacia* spp., *Banksia* spp., shrubs, sedges and forbs (Duff et al., 2013). These woodlands occur on sandy, nutrient‐poor soils, displaying deficient growth and decomposition rates (Cheal, 2010). The elevation above sea level lies between 75 and 131 m. The region's climate is cool temperate with warm summers and cool to cold winters with a mean annual rainfall of 647.9 mm and mean annual maximum and minimum temperatures of 20.2 and 8.3°C, respectively (Bureau of Meteorology, 2018). The area is ideal for studying the effects of fire and resource use on fauna as the native vegetation is highly flammable and has been subjected to prescribed burns and wildfires in the past, creating a range of habitats varying in the time since the last fire.

We used a GIS layer accessed from the local land management agency to define four temporal categories representing time since the last fire: renewal/juvenility 0–2.5 years, adolescence 2.5–10 years, maturity 10–35 years, and waning/senescence 35+ years after fire. Within these areas we used a number of criteria to define specific locations for study: (a) to remove the potentially confounding effect of vegetation type, we only considered areas classified as heathy stringybark woodland, (b) to reduce edge effects, we only used patches >20 ha, (c) whenever possible we only used areas that had been burnt once during the last 40 years (the extent of accurate records) to reduce the potentially confounding effect of fire frequency, and (d) we selected sites across the study area using a restricted random protocol across the range of postfire growth stages. Each site was set up to be 1 km apart to promote independence and 200–500 m away from vehicle tracks to reduce disturbances. A 200 m transect was established on a random bearing for small mammal trapping.

### 2.2 Mammal surveys and scat collection

Trapping was carried out in the Austral summer (December 2018 to February 2019) with sites selected haphazardly during this period to reduce temporal bias. Twenty‐five small Elliott traps (33 cm × 10 cm × 10 cm) were placed approximately 8 m apart along the transect. A piece of unbleached cotton was placed in the trap as insulation, and the trap was covered with a plastic bag to protect animals from moisture. Traps were baited with a mixture of oats, peanut butter, golden syrup, and pistachio essence, which has been shown to be useful for capturing a wide range of small mammals (Paull et al., 2011). Traps were checked between sunrise and 10 am and closed during the middle of the day to reduce stress and by‐catch. Trapping was carried out over five consecutive nights except when interrupted by adverse weather. Individuals caught were identified to species level, weighed, head‐body and tail length measured, sexed, and identified as juveniles or adults. Individuals were given a unique identifiable mark on the base of the tail using liquid paper to identify recaptures, then individuals were released at the site of capture. Occupied traps were replaced with clean traps.

Elliott traps were checked for scats each morning, with scats collected on the first capture of each individual of the target species using a pair of sterilized tweezers and stored in 5 ml vials containing ~99% ethanol. The driest pellets were selected (as an indication of the earliest defecation upon capture and least likely to contain digested bait), and scats with visible bait contamination were avoided. Each vial was given an identifier tag corresponding to the animal and was stored below 4°C until analysed.

### 2.3 Sample processing

Scat samples were analysed by eDNA Frontiers, Curtin University, Western Australia. Initially, the ethanol was removed from each vial and samples were left overnight, packed in ice in a fume cabinet allowing to evaporate the remaining ethanol. Following the evaporation, scats were cut in half and weighed. Half of each scat was processed to extract DNA, and the remainder was stored at −20°C. DNA was extracted using a Qiagen Powerfecal Pro kit, following the manufacturer's instructions and eDNA frontiers laboratory standard operating procedures, and extraction controls (*n* = 6) were included to detect the presence of cross‐contamination. Quantitative PCR (qPCR) was done at three concentrations for all extractions (neat, 1/10 dilution, and 1/100 dilution) to see whether samples exhibited inhibition and to determine optimal DNA input for PCR, for each sample to maximize input relative to any inhibitors (Murray et al., 2015). Two assays were used in this study to target invertebrates and plants. For the detection of arthropods (in this study defined as Insecta and Arachnida), the assay ZBJ‐ArtF1c/R2c was used; this assay targets a highly variable region in the cytochrome c oxidase I (COI) gene from the mitochondria DNA 16S rRNA gene (Zeale et al., 2011). For plants, we used the trnlg/h primers (Taberlet et al., 2007), which target the chloroplast trnL (UAA) intron (Taberlet et al., 2007). We did not include an assay for fungi as scat samples were collected in summer when the likelihood of feeding on sporocarps is low (Braithwaite et al., 1978; Cheal, 1987; Di Stefano et al., 2014; York et al., 2022).

The qPCRs were run on a StepOne Plus (Applied BioSystems) real‐time qPCR instrument with the following conditions: 5 min at 95°C, 40 cycles of 95°C for 30 s, 30 s at 52°C, and 45 s at 72°C, a melt curve stage of 15 s at 95°C, 1 min at 60°C, and 15 s at 95°C, ending with 10 min elongation at 72°C. The PCR mix for quantitation had a 25 μl volume and contained: 2 mM MgCl2 (Applied Biosystems), 1× PCR Gold buffer (Applied Biosystems), 0.25 mM dNTPs (Astral Scientific), 0.4 mg/ml bovine serum albumin (Fisher Biotec), 0.4 μmoL/L forward and reverse primer, 1 U AmpliTaq Gold DNA polymerase (Applied Biosystems), 0.6 μl of a 1:10,000 solution of SYBR Green dye (Life Technologies), and 2 μl of DNA template. Extraction controls, nontemplate controls, and positive controls were included for all PCR runs.

After optimal DNA input was determined by qPCR, each sample was assigned a unique combination of multiplex identifier (MID) tags for each primer assay. These MID tags were incorporated into fusion‐tagged primers, and none of the primer‐MID tag combinations had been used previously in the laboratory to prevent cross‐contamination. Fusion PCRs were done in duplicate and to minimize PCR stochasticity, the mixes were prepared in a dedicated clean room before DNA was added. The PCRs were done with the same conditions as the standard qPCRs described above, although with 50 cycles performed and the melt curve analysis omitted. Samples were then pooled into approximately equimolar concentrations to produce a PCR amplicon library that was size‐selected to remove any primer‐dimer that may have accumulated during fusion PCR. Size selection was performed (160–400 bp) using a PippinPrep 2% ethidium bromide cassette (Sage Science). Libraries were cleaned using a QIAquick PCR Purification Kit (Qiagen) and quantified using Qubit Fluorometric Quantitation (Thermo Fisher Scientific). Single‐end sequencing was performed on the Illumina MiSeq platform using the 300‐cycle V2 as per the manufacturer's instructions.

Bioinformatic tools were used to analyse raw sequence data. Results were demultiplexed and trimmed using Obitools and quality filtered with USEARCH v11 for sequencing errors; (maxee = 1) and minimum length (COI minlength = 135, trnl minlength = 30). Sequences were then dereplicated, and unique sequences were transformed into zero radius operational taxonomic units (ZOTUs) to provide sensitive taxonomic resolution (USEARCH v11; Edgar, 2018). ZOTUs, in contrast to operational taxonomic units (OTUs), are a more exact sequence variant. Generated ZOTUs were queried against the nucleotide database NCBI (GenBank) and assigned to the species level. The taxonomic assignment was based on an eDNA Frontiers in‐house python script (Mousavi‐Derazmahalleh et al., 2021), which further filters NCBI Blast results (*e* value ≤1*e*−5, %identity ≥90 and qCov ≥100), combines it with ZOTU table results, and produces a table containing the taxonomic information available from the Blast taxonomy database (accessed February 2020). The final table was curated to remove singleton assignments, duplicate taxa, nontarget taxa (not targeted by assay), and taxa found in the bait and cotton in Elliott traps (*Avena* sp. and *Gossypium* sp.). Three nonarthropod taxa were detected by the COI insect assay (yellow‐footed antechinus, a slug *Ambigolimax valentinanus*, and a nematode *Rhabditda* sp.) and they were removed from the results. The COI insect assay was developed to detect specific arthropod taxa, thus any detections of noninsect taxa using this assay are possibly caused by errors in the NCBI database or misassignment of tag sequences (tag jumping). The PCR controls were not opened in the laboratory where DNA was added, so the presence of sequence reads may be due to tag jumping. Tag jumping can occur during the sequencing step as a result of the mixed cluster on the flow cell (Kircher et al., 2012; Schnell et al., 2015). The presence of misassigned tags to the extraction controls and one of the PCR controls was very low compared with the overall number of sequence reads obtained, and it is not considered to have affected the results of the study. In eDNA metabarcoding results, the number of sequence reads obtained does not represent the abundance of particular ZOTUs present in the sample (Deagle et al., 2018; Verkuil et al., 2020) thus all data were converted to presence/absence before further analysis and ZOTUs that were present in the extraction controls were removed from the dataset.

### 2.4 Data analysis

We first created a dissimilarity matrix (Jaccard index) using the ZOTU presence/absence and individual mammal data and analysed the matrix using nonmetric multidimensional scaling (NMDS) in the R package “vegan” (Oksanen et al., 2020). NMDS results were graphically represented as biplots, showing the placement of major groups (species/growth stages) relative to each other in ordination space. Due to the small sample size in the youngest age class (5 sites), we combined the 0.5–2.5 years and 2.5–10 years postfire categories, resulting in three postfire growth stages changing the terminology to recent (0.5–10 years postfire), mid (11–35 years postfire), and late (35+ years postfire) for further analysis. Diet data were then analysed using two‐way permutational analysis of variance, PERMANOVA (Anderson et al., 2008), with species and growth stage as fixed factors, to test for differences in composition and any interaction between the factors. To further explore the potential effect of the growth stage, separate one‐way PERMANOVAs were then undertaken for each species independently. If significant effects were detected, then pairwise tests were carried out between levels of each factor. ZOTUs contributing to observed patterns of similarity/dissimilarity between groups were identified using Similarity Percentages, SIMPER (Clarke, 1993) analysis in PRIMER 7 (Clarke & Gorley, 2016). Results for these analyses are presented and analysed as the sum of the frequency of occurrence across individuals within each mammal species (hereafter denoted as frequency).

## 3 RESULTS

From the Elliott trapping carried out over 15,750 trap nights (126 sites × 25 traps × 5 nights), we captured 153small mammals (in 57 sites out of 126 sites) belonging to 10 species from three Orders (Rodentia, Diprotodontia, and Dasyuromorphia). We collected 122 scat samples (yellow‐footed antechinus; *n* = 42, bush rat; *n* = 49 and heath mouse; *n* = 31; Appendix 1: Table A1). The plant assay trnLg/h detected 102 plant taxa belonging to 5 Classes, 34 Orders, and 55 Families. Only 27 named species could be identified due to the poor taxonomic resolution of trnLg/h and/or incomplete barcode database. There were 62 arthropod taxa detected using the ZBJ‐ArtF1c/R2c assay comprising 3 Classes, 7 Orders, 26 Families, and 44 Genera. Forty‐five taxa could be reliably assigned to recognized species.

**FIGURE 1:**
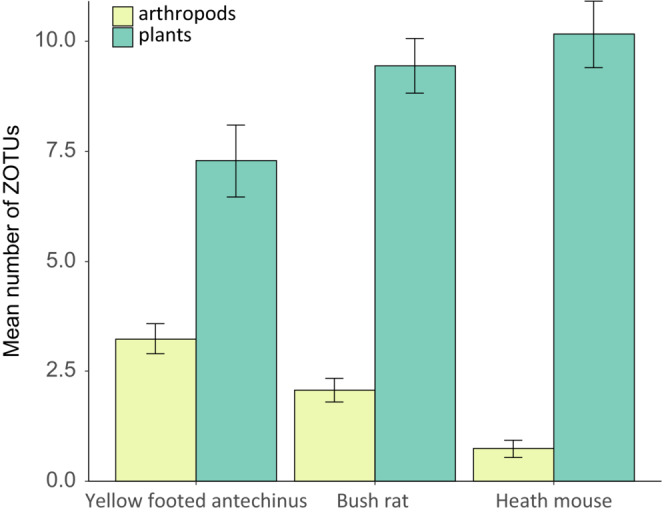
Mean number of ZOTUs per species that was detected from the COI and trnL metabarcodes. Error bars represent the Standard Error.

For the yellow‐footed antechinus, 92 food items were detected (38 arthropod species and 54 plant species; Appendix 1: Table A3), for the bush rat, 101 food items were detected (36 arthropods and 65 plants; Appendix 1: Table A4), and for the heath mouse 77 food items (15 arthropods and 62 plants; Appendix 1: Table A5). The mean number of food items per scat was similar for all species: bush rat 11.5, yellow‐footed antechinus 10.9, and heath mouse 10.5 (Figure 1, Appendix 1: Table A1). For all three species some of the scat consisted only of plant species (*n*_(YFA)_ = 3, *n*_(BR)_ = 9, *n*_(HM)_ = 15) while no arthropod‐only samples were detected.

**FIGURE 2:**
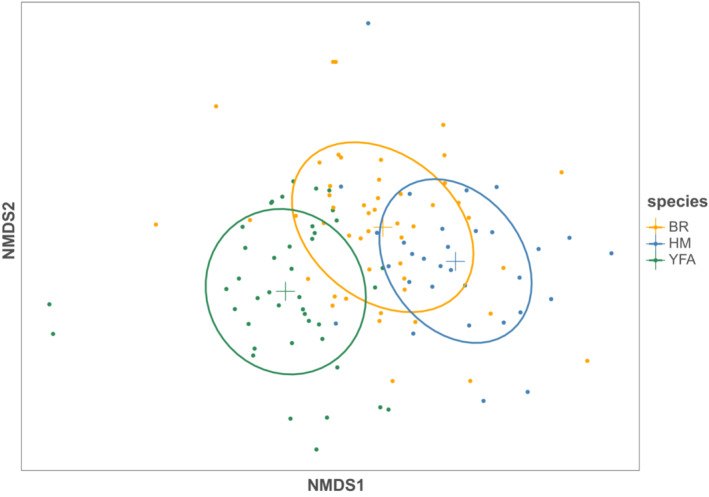
Two‐dimensional diagram representing the diets of the three species bush rat (BR, *n* = 49), heath mouse (HM, *n* = 31) and yellow‐footed antechinus (YFA, *n* = 42). Elipses represent the 95% confidence interval around the centroid for each species.

**FIGURE 3:**
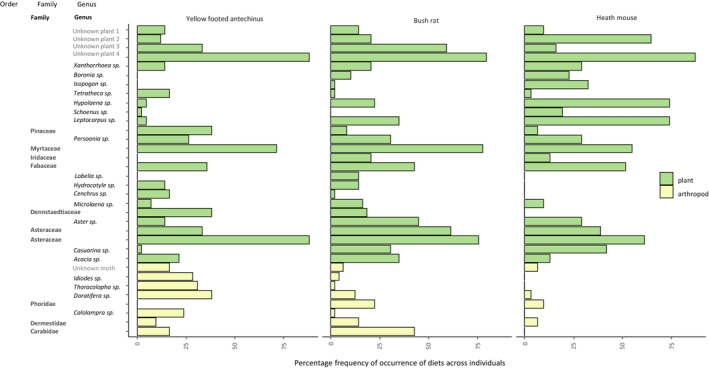
Percentage frequency of occurrence of diets of the major food species across individuals (scat samples) for each COI (arthropods) and trnL (plants) metabarcodes.

The two‐dimensional representation of NMDS results (Figure 2) and pairwise comparison of diets (Table 1) suggests gross differences in overall diet between the three species, with differences among postfire vegetation growth stages for each species less pronounced (Figure 3). While 2D stress values are relatively large, 3D representations (with lower stress) did not improve clarity, so we have used the 2D diagrams for simplicity. Two‐way PERMANOVA results indicated a significant effect of species (pseudo‐*F* = 5.8, *p* < .001, *df* = 2, *r* ^2^ = .08) and vegetation growth stage (pseudo‐*F* = 1.6, *p* < .001, *df* = 2, *r* ^2^ = .02), and a significant interaction between these two factors (pseudo‐*F* = 2.34, *p* < .001, *df* = 4, *r* ^2^ = .06).

**TABLE 1:** Pairwise comparison of diets (ZOTUs) from PERMANOVA for the three small mammal species and for the three growth stages.

Pairs	*df*	Sums of squares	Pseudo‐*F*	*R* ^2^	*p*
Pairwise comparison of diets for each species					
Yellow‐footed antechinus vs bush rat	1	1.87	7.70	.08	.001**
Yellow‐footed antechinus vs heath mouse	1	2.96	12.58	.15	.001**
Bush rat vs heath mouse	1	1.57	6.83	.08	.001**
Pairwise comparison of diets for each growth stage					
Mid vs late	1	0.66	2.57	.031	.003**
Mid vs recent	1	0.57	2.15	.029	.023*
Late vs recent	1	0.33	1.24	.01	.211

Significance codes: ^**^0.01; ^*^0.05.

### 3.1 Diets of the three species

**FIGURE 4:**
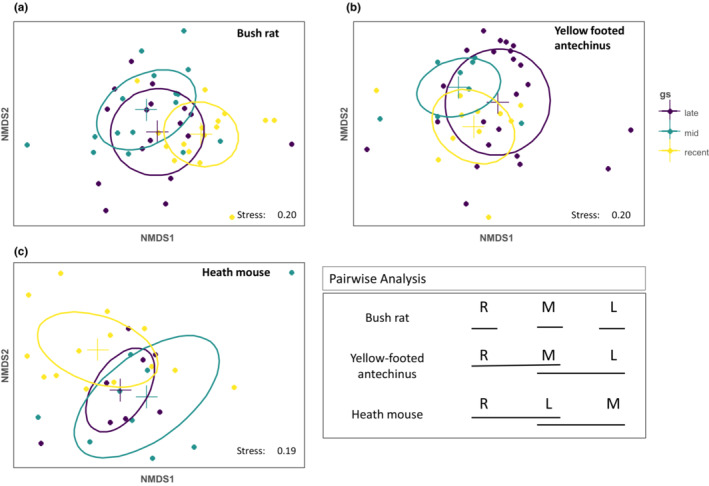
Two‐dimensional ordination diagram representing the diets of the three species separately for three post‐fire growths stages, R = recent (0.5 to 10 years post‐fire), M = mid (11 to 35 years post‐fire) and L = late (35+ years post‐fire). Elipses represent the 95% confidence for each species. In the pairwise analysis box, continuous lines that connect letters indicate no significant difference between levels of the factor (see Appendix 1: Table A2).

The yellow‐footed antechinus and bush rat consumed a diverse array of plants and arthropods, while heath mouse diets consisted predominantly of plants and comparatively a low number of arthropod taxa (Figure 4). Of the three species, the yellow‐footed antechinus had a higher frequency of occurrence of arthropod food taxa across individuals while, for other two mammal species, plants were found in the higher frequency of occurrence across individuals. However, there was a considerable variation among the diets of individuals within species.

The yellow‐footed antechinus arthropod food items consisted of 37 insect taxa (moths, beetles, cockroaches, flies, lacewings) and a species of spider (*Cheiracanthium* sp.). Moths (Order Lepidoptera) constituted the bulk of the dietary ZOTUs, with the painted cup moth *Doratifera oxleyi* the most frequently detected, followed by two bracken moths from the family Geometridae (*Idiodes siculoides* and *Idiodes apicata*) and *Thoracolopha spilocrossa* from the family Noctuidae (Appendix 1: Table A3). A species of cockroach, (*Calolampra* sp.), was also frequently detected. Overall, 54 species of plants were detected (myrtles, pines, ferns, daisies). Species belonging to Myrtaceae, Fabaceae (*Acacia* sp. and *Kennedia* sp.), Asteraceae, and Proteaceae were among those detected at the highest frequency.

Overall, bush rat arthropod food items consisted of 35 insect taxa (moths, flies, beetles) and a crustacean (yabbie). The most frequently detected insect was a species of carabid beetle, additionally beetles belonging to the family Dermestidae, dipterans belonging to the family Phoridae, and family Chironomidae (nonbiting midges, *Polypedilum* sp.) were detected at high frequencies. A wide variety of moths were detected in low frequencies. Overall, plants consisted of 65 species with unidentified plant ZOTU162 being the most frequently detected. Species belonging to the families (Myrtaceae, Asteraceae, Fabaceae, and *Acacia* sp., *Pultenaea* sp.). *Leptocarpus* sp., *Hypolaena* sp., and *Centrolepis monogyna* were found at the highest frequencies. A wide variety of other plant species was found at low frequencies (Appendix 1: Table A4).

Overall, heath mouse arthropod food items consisted of 14 taxa including moths, beetles, flies, and spiders. The most frequent insect detected was the same species of carabid beetle identified in the other two small mammals, with other taxa detected at low frequencies. A large number of plant species belonging to a broad range of families was detected in heath mouse scats, comprising 62 plant taxa (primarily rushes, Gentianales, and myrtles). The most frequent plant species was an unidentified plant ZOTU162. Heath mouse scats contained a high frequency of occurrence of plants belonging to the rushes of the family Restionaceae (*Hypolaena* sp., *Leptocarpus* sp.; Appendix 1: Table A5).

Differences in diet between species were a consequence of cumulative small differences across a range of ZOTUs. While SIMPER results (Table 2) reflect some overlap in diet items, it is clear that species have, overall, distinctive diet items based on variations in their frequency of occurrence across individuals. Based on the results of the SIMPER analysis, the highest contribution to the difference in the diets of three species is given by a Fabaceae plant species (ZOTU99) and a Myrtaceae plant species (ZOTU115) that were not able to be classified beyond the class level. A Magnoliopsida (ZOTU154) plant contributed to the highest dissimilarity between yellow‐footed antechinus and bush rat. Additionally, a plant species belonging to the family Pinaceae (most likely *Pinus radiata*) contributed to the dissimilarity among yellow‐footed antechinus and the other two species. Only two insect species the painted cup moth (*Doratifera oxleyi*) and a beetle species belonging to the family Carabidae contributed substantially to the dissimilarity between yellow‐footed antechinus and other two species, although yellow‐footed antechinus is considered an insectivore. An unidentified plant species (ZOTU154) and a daisy belonging to the family Asteraceae (ZOTU78) contributed to the highest dissimilarity among bush rats and other two species. Plant species belonging to the family Rubiaceae and a rush (*Hypolaena* sp.) contributed to the highest dissimilarity between heath mice and other two species. The plant ZOTU162 (unidentified plant species) also contributed to the dissimilarity of diets between heath mice and other two species.

**TABLE 2:** Mean frequency of occurrence of ZOTUs identified by similarity percentages (SIMPER) as contributing to the overall compositional dissimilarity between the three mammal species fire).

ZOTU	Family/Genus/Species name (if identified)	Average frequency of occurrence			Percentage contribution to pairwise dissimilarity		
		YFA	BR	HM	YFA‐BR	YFA‐HM	BR‐HM
162	Plant (Unidentified)	0.88	0.80	0.87	2.5	1.9	2.3
114	Myrtaceae	0.88	0.76	0.61	2.7	3.3	3.3
115	Myrtaceae	0.71	0.78	0.55	3.1	3.5	3.5
28	*Doratifera oxleyi*	0.38	0.12	0.03	2.9	2.7	0.8
160	Dennstaedtiaceae	0.38	0.18	0.00	2.7	2.3	1.1
157	Pinaceae	0.38	0.08	0.06	2.3	2.2	0.8
78	Asteraceae	0.33	0.61	0.39	3.9	2.9	3.7
154	Plant (Unidentified)	0.33	0.59	0.16	3.8	2.1	3.8
99	Fabaceae	0.36	0.43	0.52	3.4	3.4	3.5
138	*Leptocarpus* sp.	0.05	0.35	0.74	2.4	4.7	4.0
77	*Aster* sp.	0.14	0.45	0.29	3.3	2.2	3.3
104	Rubiaceae	0.12	0.20	0.65	1.7	4.3	4.1
100	*Casuarina* sp.	0.02	0.31	0.42	2.1	2.6	3.1
4	Carabidae	0.17	0.43	0.13	3.0	1.7	2.9
140	*Isopogon anemonifolius*	0.00	0.02	0.32	0.1	1.9	2.0
137	*Hypolaena* sp.	0.05	0.22	0.74	1.7	4.6	4.2

Abbreviations: BR, bush rat; HM, heath mouse; YFA, yellow‐footed antechinus.

### 3.2 Diet change over time after fire

Species‐specific PERMANOVA results indicated an effect of postfire growth stage for all three species (bush rat; pseudo‐*F* = 2.5, *p* < .001, yellow‐footed antechinus; pseudo‐*F* = 1.5, *p* < .017, heath mouse; pseudo‐*F* = 1.4, *p* < .022). The diet of yellow‐footed antechinus changed incrementally from the recent to the late postfire growth stage, with a statistically significant difference between recent and late growth stages (*p* = .013; Figure 3, Appendix 1: Table A2). By contrast, the bush rat diet was significantly different among all growth stages (recent vs late—*p* = .002, recent vs mid—*p* = .001, late vs recent—*p* = .023; Figure 3, Appendix 1: Table A2). Pairwise analysis results indicated that the heath mouse diet in the mid postfire growth stage differed from the recent postfire growth stage (*p* = .025; Figure 3, Appendix 1: Table A2). Again, differences in diet between growth stages for each species were a consequence of cumulative small differences across a range of ZOTUs. While SIMPER results (Tables 3, 4, 5) reflect some overlap in diet items, it is clear that species have, overall, distinctive diet items based on their frequency of occurrence across individuals.

**TABLE 3:** Mean frequency of occurrence of ZOTUs identified by similarity percentages (SIMPER) as contributing to the overall compositional dissimilarity between growth stages for the yellow‐footed antechinus.

ZOTU	Family/Genus/Species name (if identified)	Average frequency of occurrence			Percentage contribution to pairwise dissimilarity		
		Recent	Mid	Late	R‐M	R‐L	M‐L
162	Plant (Unidentified)	0.91	1.00	0.83	1.0	3.2	2.1
114	Myrtaceae	0.91	0.88	0.87	2.8	2.6	2.3
115	Myrtaceae	0.73	0.88	0.65	3.9	4.4	3.4
28	*Doratifera oxleyi*	0.45	0.25	0.39	4.1	4.6	3.5
157	Pinaceae	0.36	0.38	0.39	4.2	3.9	2.9
3	*Calolampra* sp.	0.27	0.00	0.30	2.7	4.0	2.5
57	Plant (Unidentified)	0.27	0.00	0.17	2.1	2.5	0.9
4	Carabidae	0.18	0.50	0.04	4.9	1.8	3.7
141	*Persoonia* sp.	0.18	0.38	0.26	3.5	2.4	2.9
35	*Thoracolopha spilocrossa*	0.18	0.00	0.43	1.7	3.3	2.3
78	Asteraceae	0.00	0.50	0.43	3.9	2.6	3.4
23	*Idiodes siculoides*	0.09	0.38	0.39	4.7	2.5	3.8
66	*Hydrocotyle* sp.	0.09	0.38	0.09	3.2	0.9	2.5
160	Dennstaedtiaceae	0.09	0.25	0.57	2.8	4.4	3.7
99	Fabaceae	0.09	0.38	0.48	2.9	4.0	3.5
154	Plant (Unidentified)	0.09	0.38	0.43	3.3	3.0	3.1

**TABLE 4:** Mean frequency of occurrence of ZOTUs identified by similarity percentages (SIMPER) as contributing to the overall compositional dissimilarity between growth stages for the bush rat.

ZOTU	Family/Genus/Species name (if identified)	Average frequency of occurrence			% Contribution to pairwise dissimilarity		
		Recent	Mid	Late	R‐M	R‐L	M‐L
78	Asteraceae	0.88	0.44	0.53	4.2	3.8	3.4
154	Plant (Unidentified)	0.88	0.38	0.53	4.4	3.9	3.3
77	Asteraceae	0.75	0.38	0.24	4.1	5.0	2.7
114	Myrtaceae	0.81	0.69	0.76	2.9	3.0	2.9
115	Myrtaceae	0.75	0.81	0.76	2.6	3.1	2.8
162	Plant (Unidentified)	0.75	0.75	0.88	2.9	2.8	2.4
6	Dermestidae	0.31	0.06	0.06	2.0	2.1	0.6
75	Iridaceae	0.00	0.63	0.00	4.1	0.0	4.1
100	*Casuarina* sp.	0.06	0.63	0.24	3.9	2.0	3.7
4	Carabidae	0.31	0.56	0.41	3.4	3.5	3.4
93	*Acacia* sp.	0.25	0.50	0.29	3.2	2.9	3.3
141	*Persoonia* sp.	0.19	0.50	0.24	3.2	2.2	3.2
99	Fabaceae	0.31	0.38	0.59	3.2	4.1	3.5
138	*Leptocarpus* sp.	0.13	0.38	0.53	2.5	3.6	3.3
137	*Hypolaena* sp.	0.13	0.13	0.41	1.3	3.1	2.8

**TABLE 5:** Mean frequency of occurrence of ZOTUs identified by similarity percentages (SIMPER) as contributing to the overall compositional dissimilarity between growth stages for the heath mouse.

ZOTU	Family/Genus/Species name (if identified)	Average frequency of occurrence			% Contribution to pairwise dissimilarity		
		Recent	Mid	Late	R‐M	R‐L	M‐L
104	Rubiaceae	0.93	0.33	0.50	5.4	4.2	3.8
162	Plant (Unidentified)	0.86	0.89	0.88	2.1	2.0	2.0
138	*Leptocarpus* sp.	0.71	0.78	0.75	3.3	3.4	3.2
137	*Hypolaena* sp.	0.64	0.67	1.00	3.6	3.5	3.1
99	Fabaceae	0.57	0.33	0.63	3.8	4.0	4.3
140	*Isopogon anemonifolius*	0.43	0.11	0.38	3.0	3.7	2.9
100	*Casuarina* sp.	0.43	0.22	0.63	3.1	4.2	4.3
114	Myrtaceae	0.43	0.78	0.75	4.3	4.7	3.0
115	Myrtaceae	0.36	0.67	0.75	4.0	4.9	3.5
74	*Xanthorrhoea* sp.	0.29	0.44	0.13	3.2	2.4	3.2
77	Asteraceae	0.29	0.33	0.25	3.3	3.0	3.4
78	Asteraceae	0.43	0.33	0.38	3.5	3.7	3.6
141	*Persoonia* sp.	0.36	0.11	0.38	2.4	3.8	3.4
91	*Tetracera* sp.	0.07	0.00	0.38	0.5	3.0	2.8

For the yellow‐footed antechinus, the plant species that contributed most strongly to the pairwise dissimilarities among growth stages were unidentified plant species ZOTU162, Myrtaceae plant species ZOTU115, Asteraceae species ZOTU78. Additionally, a bracken moth species (*Idiodes siculoides*) and a cockroach species (*Calolampra* sp.) contributed to this diet difference. For the bush rats, both plants and arthropods contributed to the dissimilarities between the diets of the three growth stages. Some of the prominent contributions to this dissimilarity were Asteraceae species, Magnoliopsida species, and a beetle species belonging to the family Dermestidae. For the heath mouse, a plant species belonging to the family Rubiaceae and *Tetracera* sp. showed the highest percent contribution to the difference between growth stages. Arthropods did not contribute to this difference among growth stages for the heath mouse.

## 4 DISCUSSION

It is important to understand how diet is influenced by disturbances such as fire as this will help conservation management in future when fires are predicted to be larger and more frequent (Flannigan et al., 2000). Our focal species: the yellow‐footed antechinus, the bush rat, and the heath mouse, have been studied extensively in other ecological aspects such as genetics, population structure, landscape, and fire ecology (Cockburn et al., 1981; Marchesan & Carthew, 2004; Nalliah et al., 2021; Smith, 1984). However, there is a paucity of in‐depth information on their diets. In this study, we used fecal eDNA metabarcoding to determine differences in the diets of these three small mammal species and changes in their diets over time after fire. Our focus was on longer‐term effects, with sites ranging from 1–79 years since last burnt. We obtained a robust set of data on diets at a high taxonomic resolution. eDNA metabarcoding allowed new insight into the dietary patterns of these species, such as the yellow‐footed antechinus consuming a wide variety of plants and moths. Overall, the diets of the three species were fundamentally different from each other, as was the nature of diet changes after a fire. The bush rat showed the most pronounced diet changes throughout the postfire vegetation growth stages, while the heath mouse showed the least.

### 4.1 Diet of the three species

The rate of passage of food in small mammals is fast, generally within few hours of the food intake (Karasov et al., 1986), thus detected food species detections in scat can be inferred as items ingested in a foraging bout close to the time of capture. The average number of food items per scat for individuals of all three species was similar suggesting that in a single foraging bout all three species consumed a high variety of food species from several broad types of food. Being a generalist is an advantageous foraging strategy in a fire‐prone environment where resource availability is unpredictable or variable after fires (Cruz‐Rivera & Hay, 2000; Di Stefano et al., 2014; Sutherland & Dickman, 1999).

We expected to find gross dietary differences between the three species and our results largely agree with previous work (Cheal, 1987; Di Stefano et al., 2014; Hindmarsh & Majer, 1977). Yellow‐footed antechinus and bush rats consumed a diverse array of plants and arthropods, while heath mouse diet consisted of prominently plants and comparatively a low number of arthropod taxa. Braithwaite et al. (1978) suggested five food niches for small mammals in heathland environments, and their activity patterns are directly related to diet and predation pressure. Here we can broadly classify the three species into that categorization: bush rat—omnivore, heath mouse—generalist herbivore, and yellow‐footed antechinus—scansorial insectivore; however, our results indicate that yellow‐footed antechinus diet shows considerable plasticity compared to that of an exclusive insectivore.

Surprisingly, there were many plants detected in the diet of yellow‐footed antechinus in contrast to earlier microscopic work that found only invertebrates and vertebrates (Hindmarsh & Majer, 1977). One likely explanation for this is that the metabarcoding analysis is detecting plant products such as nectar, pollen, and plant sap that would not be detected in microscopic analysis. There have been records of yellow‐footed antechinus feeding on the nectar of *Banksia* flowers and sap of the Sticky Hop Bush *Dodonaea viscosa* (Goldingay, 2000; McCreadie, 2017); similarly, we suspect that yellow‐footed antechinus could be feeding on the sap of pine wildlings (*Pinus radiata*), which was frequently recorded. Our findings on plant matter are further confirmed by a micro‐histological study (York et al., 2022) carried out in the same landscape indicating that there is a considerable amount of plant matter such as leaves, seeds, and flowers in yellow‐footed antechinus diet. Thus, we suggest that this species is not solely insectivorous, and individuals often supplement their diet with plant material. Many diet studies have reported plant material in carnivore and insectivore stomach content and scats (Yoshimura et al., 2021). Carnivorous mammals such as foxes, badgers civets, and insectivorous mammals such as aardvarks, and bats are reported to seasonally augment their diets with plant material such as berries, roots, nectar, and in some instances, foliage (Frick et al., 2014; Koike et al., 2008; Milton & Dean, 2001; Mudappa et al., 2010). Berries, nectar, and seeds contain high carbohydrate levels, amino acids, and other micronutrients (Ball & Golightly, 1992; Venjakob et al., 2022) substantially contributing to an animal's nutritional requirements. Furthermore, acquiring nutrient‐rich sessile plant material while actively foraging for arthropod prey could be an energy‐optimizing strategy. Thus, utilizing supplementary plant material in order to survive in a patchy resource environment could be a strategy that helps antechinus complete its dietary requirements while looking for arthropod prey.

We recorded more lepidopterans in the diet of yellow‐footed antechinus than in the previous studies (Kelly, 2006; Lada, Thomson, et al., 2008). The difference could be due to the soft body parts of lepidopterans remaining undetected in micro‐histological analysis. Due to the nature of the metabarcoding results, it was not possible to conclude which developmental stage (eggs, larvae, pupae, or adults) of moths yellow‐footed antechinus preyed on. Being nocturnal and scansorial in nature, we assume they could be foraging on resting adult moths (Kawahara et al., 2017). For the moth species that were detected in the highest frequency, *Doratifera oxleyi* (painted cup moth), the larval host plants are *Eucalyptus* trees, the dominant canopy species at our sites. For two bracken moth species detected at a high frequency (*Idiodes siculoides* and *Idiodes apicata*) larval host plants included ferns such as *Pteridium esculentum*, a common element of the heathy woodland understory. Semi‐arboreal nature of yellow‐footed antechinus is reflected in the diet with the presence of both arboreal and understory‐dwelling arthropods. Similar to earlier records (Hindmarsh & Majer, 1977), cockroaches were found in the diet of several individuals. Although we expected to see more Arachnida in the diets of yellow‐footed antechinus (see Dickman, 1986; Dickman & Happold, 2022; Hindmarsh & Majer, 1977), there was only a single record of that Class. However, in our study sites arachnids were found abundantly based on the invertebrate surveys; it is possible that arachnida species that yellow‐footed antechinus prey on are sparsely represented in our study sites or underrepresented by the eDNA metabarcoding because of mismatches between the arthropod primers and template sequence. The assays were not designed to detect vertebrate prey items; thus, there is a possibility that diet information on vertebrates is lacking in our set of results. We suspect that this could be the case for one yellow‐footed antechinus scat for which we did not detect any food items. We assume that this individual recently consumed a diet of vertebrates and as such was not identified in the metabarcoding process.

For the bush rat, our observations were similar to that reported in the literature, where rats are following an opportunistic foraging pattern (Carron et al., 1990; Cheal, 1987). This could be an indicator that bush rats have the ability to adjust their diets according to the resource availability and successfully utilizing the most optimal strategy of foraging according to different resources available (Dickman & Happold, 2022; Kelt et al., 2004). Bush rat diets consisted of the highest number of unique ZOTUs (*n* = 101), including different types of arthropods and plants, making its diet more diverse compared with the other two species. Some of the major arthropod food types found in bush rat diets were ground beetles (family Carabidae) and skin beetles (family Dermestidae), which feed on dry and dead plant materials. Furthermore, a wide variety of moths were recorded in bush rat diets. This dietary flexibility is most likely one of the reasons for its large geographic distribution. Given that bush rats have been reported consuming considerable amounts of fungi in wet sclerophyll forests in south‐eastern Australia (Vernes et al., 2015) their diet in these drier woodlands may be even broader than the metabarcoding results suggest.

Compared with other two species, the heath mouse diet consisted predominantly of plant species and a very low number of arthropods. Plants of family Restionaceae, which include *Hypolaena* sp. (possibly *Hypolaena fastigiate—*a common species in heathlands of the region (Duff et al., 2013)), and *Leptocarpus tenax*, which are found in wet soils and seasonal swamps, formed a major component of their diet. Heath mice were generally confined to vegetation with a very dense understory such as wet heaths and prefer floristically diverse habitats (Nalliah et al., 2021), Our finding suggests that these species are consuming the plant species that are commonly found in wet heath and confirms that this species can be classified as a generalist herbivore within the specific habitat in which it occurs.

### 4.2 Effect of postfire growth stage on diet

After a fire, habitats often progress through a series of successional stages where productivity and species composition change over time (Smith, 2018). Thus, fire changes the distribution and abundance of numerous resources including plants and arthropods that are commonly consumed by small mammals (Dickman & Happold, 2022; Fox, 1982; Kelly et al., 2011; Pulsford et al., 2014). Luo and Fox (1994) found that the diet of the eastern chestnut mouse *Pseudomys gracilicaudatus* varied with the postfire successional stage; initially, consuming a high proportion of leaf material, then stem, seeds, fungi, and insects, then as vegetation matured into the old stage, the composition of seeds, fungi, and insects was reduced. At the landscape scale, multiple postfire ages are often available for small mammal species, providing a greater diversity of habitats and potential food items (Jones & Tingley, 2021; Kelly et al., 2017). Different dietary strategies may make species more or less vulnerable to postfire changes and can potentially influence the ability of species to exploit the resources available in a mosaic of postfire ages.

Here, we found that the three species shifted their diets in response to postfire growth stage, with this pattern being most pronounced for the bush rat and least for heath mice. This may be because bush rats are generalist omnivores that can shift their diet in response to the local abundance of resources. In this study, they frequently consumed plants of the genus Asteraceae in the recently burnt growth stage. This could indicate that they are feeding on flowers of annual plants or small shrubs that may be locally abundant after fires, while later in the succession woody shrubs such as *Casuarina* and sedges were more commonly detected, highlighting a shift with changing resource availability. As such, dietary plasticity may allow this species to persist immediately after fires and throughout long‐term succession (Di Stefano et al., 2014; Sutherland & Dickman, 1999).

Yellow‐footed antechinus diet shifts were most pronounced when comparing recent compared with late successional stages. The plants that changed in frequency over time included plants of the families Myrtaceae and Asteraceae and this could be due to yellow‐footed antechinus targeting certain resources that may be abundant at different times in the postfire succession. Fire could change the time of seedling recruitment and the time of flowering and nectar production thus changing the resource available at different postfire ages (Benwell, 1998; Pyke, 2017). However, we were not able to identify to defined species many of the particular plant resources that were being utilized by yellow‐footed antechinus and further work needs to be done to identify the tissue types consumed by these species.

In contrast to the yellow‐footed antechinus and bush rat, the heath mouse is a generalist herbivore, thus may be more limited in their options in a change in their diets in response to changing resources. We only found a small dietary shift from early to the mid postfire age in the heath mouse. This could be because they eat common plants that are present at all stages of the succession (Di Stefano et al., 2014). For example, *Hypolaena fastigiata* is a common plant present in all the growth stages of postfire ages in treeless heath (Duff et al., 2013). Thus, the feeding strategy of heath mice appears to rely on a common plant species that are found in these areas, where they do not have to change their diets substantially in response to fire. Furthermore, wetter heath areas regenerate rapidly after fires creating ideal environments for heath mouse (Benwell, 1998). Our conclusions are well aligned with the confined distribution of these species in wet heath areas, and they are already selecting for habitats that provide them with preferred diet items. This clearly contrasts with other two species with broader distribution and more prominent change of diets in response to changing conditions in postfire environments. Determining species dietary plasticity in response to disturbance would be further enhanced by pairing a diet study with an assessment of how food availability and quality change over time (e.g., Di Stefano & Newell, 2008). While we have, general knowledge of the shifting postfire food resource base in these ecosystems (Duff et al., 2013) we lack detailed information about how the availability of different food species changes over time.

### 4.3 Metabarcoding considerations

While we were able to gain powerful insights into the diets of the three species in this study, there were examples where we could not identify common plant species beyond the class level. eDNA metabarcoding is only as good as the target taxonomic libraries that underpin them (Dormontt et al., 2018; Rathnasingham & Hebert, 2007), thus it is important to create complete metabarcoding libraries for different taxa in order to increase the accuracy of results. Metabarcoding will become even more powerful as libraries become more complete, as demonstrated by our ability to detect moths at the species level, where well‐developed libraries exist.

As the number of sequence reads obtained in eDNA metabarcoding results does not represent the abundance of particular ZOTUs present in the sample, the relative proportions (e.g., volume) of individual food species cannot be determined. As such the importance of particular food types (e.g., by volume) cannot be determined. While this places some constraints on interpretation, our results have shed new light on the breadth of food items consumed by our three target species.

Paradoxically, the power of the technique can create issues whereby secondary predation is detected in diets. Generally, when DNA‐based diet studies are carried out for omnivores, multiple makers for different taxa such as plants, arthropods, and fungi are used to obtain an understanding of the range of diets (Bonin et al., 2020; Robeson et al., 2018; Taberlet et al., 2018). In this study, we used two markers to identify plants and arthropods as our study species consume a wide variety of dietary items in both categories. Thus, there is a possibility for secondary predation to be detected in diets (Berman & Moshe, 2022; Taberlet et al., 2018; Tercel et al., 2021). For example, it is possible that some plant materials detected in yellow‐footed antechinus scat were originally eaten by invertebrates. However, we consider it likely that most of the ZOTUs in our diet results were a consequence of primary predation, as they showed similar trends as past records and earlier data from the same study area (York et al., 2022).

Animals can select highly nutritious tissues over less nutritious tissues of the same plant or animal (Deagle et al., 2010). Understanding which functional parts of plants and what larval stages of arthropods are being consumed is an important aspect of understanding small mammal‐habitat interactions. Such information can provide important insight into animal behavior such as predator–prey interactions, and ecological functions performed by species such as seed dispersal and pollination (Gende et al., 2001; Klare et al., 2011; Tercel et al., 2021). However, eDNA metabarcoding alone cannot differentiate between the tissue types that are consumed (Tercel et al., 2021). For example, micro‐histological analysis of silky mice *(P. apodemoides)* diet showed that they ate more seeds in recently burnt compared with older postfire stages (Di Stefano et al., 2014), a finding that would have been missed if metabarcoding alone had been used. For yellow‐footed antechinus, questions remain to be explored on what type of plant tissue and what developmental stage of arthropods are consumed in order to get a complete understanding of diets. Thus, incorporating micro‐histological methods with eDNA metabarcoding studies will give better insight into how animal diets are changing in response to changing environments.

## 5 CONCLUSION

Analysis of animal diets sheds light on critical ecological interactions and is beneficial in conservation management decision‐making. For example, new information on the diets of flying foxes improved the understanding of how they function as pollinators and seed dispersers (Bell et al., 2020). However, information regarding diet changes over time after disturbance is lacking for many animals living in habitats prone to disturbances such as fire. Using eDNA metabarcoding of scat samples, we obtained high taxonomic resolution data on the diet of three species and identified cryptic taxa that would have gone unnoticed if conventional dietary analysis methods were used. Although eDNA metabarcoding is a powerful biodiversity monitoring tool, in dietary analysis it cannot differentiate different tissue types such as seeds, flowers, foliage, or development stages of arthropod species, which are essential in understanding ecological interactions. The use of complementary biodiversity monitoring methods has been recommended in many eDNA metabarcoding studies (Ryan et al., 2022; Tordoni et al., 2021; Valdivia‐Carrillo et al., 2021), similarly dietary analysis can be improved in future by combining molecular and histological methods (Deiner et al., 2017; Shutt et al., 2020).

From a land management perspective, this study revealed important information about changes in diet over time after fire, highlighting differences among the three species, which reflect their life‐history strategies. Through the identification of critical resources, appropriate conservation management actions can be undertaken to protect and augment such resources. For example, in this woodland ecosystem, after the critical nesting (hollows) and food (seed) resources for the south‐eastern red‐tailed black cockatoo (*Calyptorhynchus banksii graptogyne*) were identified, appropriate conservation measures have been implemented to sustain these endangered bird populations (Maron et al., 2008). The three species studies here do not specialize in individual food sources; however, information gleaned from metabarcoding can be used to guide future research and management. For example, determining how the quality and quantity of important food sources, such as the sedges for heath mice and moths for yellow‐footed antechinus, are affected by variation in fire regimes will help to determine appropriate fire management actions for these species. Furthermore, determining fire management strategies that promote a diversity of food resources at both local and landscape scales would be beneficial, especially to species with broad diets such as bush rats and yellow‐footed antechinus. By shedding light on the dietary requirements of three native mammal species and changes in resource use over time after fire, this study provides guidance to land managers to conserve populations of small mammals across the landscape through appropriate fire management.

## AUTHOR CONTRIBUTIONS

**Saumya Wanniarachchi:** Conceptualization (equal); data curation (lead); formal analysis (equal); funding acquisition (equal); investigation (lead); methodology (equal); project administration (lead); visualization (lead); writing – original draft (lead); writing – review and editing (equal). **Matthew Swan:** Conceptualization (equal); formal analysis (equal); funding acquisition (lead); investigation (equal); methodology (equal); project administration (equal); supervision (lead); validation (lead); writing – review and editing (lead). **Paul Nevill:** Funding acquisition (lead); methodology (supporting); project administration (supporting); resources (lead); validation (supporting); writing – review and editing (equal). **Alan York:** Conceptualization (equal); formal analysis (equal); funding acquisition (lead); investigation (equal); methodology (lead); project administration (lead); supervision (lead); validation (lead); writing – review and editing (lead).

## CONFLICT OF INTEREST

We declare that there is no conflict of interest for this work.

## Data Availability

The data that support the findings of this study are available from the corresponding author upon reasonable request.

## APPENDIX 1

**TABLE A1:** Number of scat samples, unique number of ZOTUs and mean prey number per a scat sample for each species

Species	Scat samples	Number of unique ZOTUs	Mean prey number per a scat sample	Std. Error of mean
Bush rat (Rattus fuscipes)	49	101	11.5	0.72
Heath mouse (Pseudomys shortridgei)	31	92	10.9	0.81
Yellow‐footed antechinus (Antechinus flavipes)	42	77	10.5	1.01
All 3 species	112	155	11.0	0.49

**TABLE A2:** Pairwise analysis of the diets from PERMANOVA of three species for different post fire growth stages.

Pairs	*df*	Sums of squares	Pseudo‐*F*	*R* ^2^	*p*
Bush rat					
Late vs recent	1	0.65	3.13	.09	.002*
Late vs mid	1	0.49	2.13	.06	.023
Recent vs mid	1	1.08	5.20	.15	.001*
Yellow‐footed antechinus					
Mid vs late	1	0.36	1.45	.05	.135
Mid vs recent	1	0.32	1.51	.08	.122
Late vs Recent	1	0.53	2.19	.06	.013
Heath mouse					
Mid vs late	1	0.25	1.20	.07	.277
Mis vs recent	1	0.45	2.02	.09	.025
Late vs recent	1	0.29	1.47	.07	.115

Significance codes: ^*^0.05.

**TABLE A3:** List of food species detected for yellow‐footed antechinus scat samples.

Class	Order	Family	Genus/ Species	ZOTU ID	Sum of frequency of occurrence
Yellow‐footed antechinus (*Antechinus flavipes*) Scat samples = 42					
*List of arthropods detected from COI metabarcode (Unique ZOTUs = 38)*					
Insecta	Lepidoptera		UNKNOWN	ZOTU57	7
		Limacodidae	*Doratifera oxleyi*	ZOTU28	16
			*Doratifera* sp.	ZOTU29	2
		Geometridae	*Idiodes siculoides*	ZOTU23	13
			*Idiodes apicata*	ZOTU22	2
			UNKNOWN	ZOTU24	1
		Noctuidae	*Thoracolopha spilocrossa*	ZOTU35	12
			*Agrotis porphyricollis*	ZOTU30	6
			*Thoracolopha melanographa*	ZOTU34	5
			*Agrotis* sp.	ZOTU31	2
			*Persectania dyscrita*	ZOTU32	2
			*Proteuxoa hypochalchis*	ZOTU33	1
			UNKNOWN	ZOTU36	1
		Oecophoridae	*Prodelaca achalinella*	ZOTU43	4
			*Prodelaca* sp.	ZOTU44	4
			*Enoplidia simplex*	ZOTU37	2
			*Eomichla* sp.	ZOTU38	1
			*Garrha* sp.	ZOTU41	1
			*Heliocausta oecophorella*	ZOTU42	1
			UNKNOWN	ZOTU45	1
		Hepialidae	*Oxycanus* sp.	ZOTU26	5
		Erebidae	*Castulo doubledayi*	ZOTU16	4
			*Calamidia hirta*	ZOTU15	1
			*Praxis aterrima*	ZOTU19	1
		Psychidae	*Cebysa leucotelus*	ZOTU46	2
			*Lepidoscia retinochra*	ZOTU48	1
		Pyralidae	*Stericta carbonalis*	ZOTU51	3
			*Endotricha pyrosalis*	ZOTU49	2
		Tineidae	*Tineola bisselliella*	ZOTU53	4
		Cosmopterigidae	*Macrobathra ceraunobola*	ZOTU12	1
	Blattodea	Blaberidae	*Calolampra* sp.	ZOTU3	10
	Coleoptera	Carabidae	UNKNOWN	ZOTU4	7
		Dermestidae	UNKNOWN	ZOTU6	4
		Cleridae	*Eleale* sp.	ZOTU5	1
	Diptera		UNKNOWN	ZOTU11	1
		Phoridae	*Megaselia* sp.	ZOTU9	2
	Neuroptera	Hemerobiidae	*Micromus tasmaniae*	ZOTU58	2
Arachnida	Araneae	Miturgidae	*Cheiracanthium* sp.	ZOTU2	1
*List of plants detected from trnL metabarcode (Unique ZOTUs = 54)*					
Streptophyta			UNKNOWN	ZOTU162	37
Magnoliopsida			UNKNOWN	ZOTU154	14
	Myrtales		UNKNOWN	ZOTU114	37
		Myrtaceae	UNKNOWN	ZOTU115	30
	Fabales	Fabaceae	UNKNOWN	ZOTU99	15
			*Acacia* sp.	ZOTU93	9
			*Kennedia* sp.	ZOTU95	2
	Asterales	Asteraceae	UNKNOWN	ZOTU78	14
			*Aster* sp.	ZOTU77	6
	Proteales	Proteaceae	*Persoonia* sp.	ZOTU141	11
			UNKNOWN	ZOTU133	1
	Oxalidales	Elaeocarpaceae	*Tetratheca* sp.	ZOTU117	7
		Oxalidaceae	*Oxalis* sp.	ZOTU118	2
	Poales	Poaceae	*Cenchrus* sp.	ZOTU129	7
			*Microlaena* sp.	ZOTU132	3
			*Stipa* sp.	ZOTU134	2
			UNKNOWN	ZOTU135	2
			*Holcus* sp.	ZOTU130	1
			*Rytidosperma* sp.	ZOTU133	1
		Restionaceae	*Hypolaena* sp.	ZOTU137	2
			*Leptocarpus* sp.	ZOTU138	2
		Cyperaceae	*Lepidosperma tortuosum*	ZOTU120	1
			*Schoenus apogon*	ZOTU124	1
	Apiales	Araliaceae	*Hydrocotyle* sp.	ZOTU66	6
		Apiaceae	UNKNOWN	ZOTU65	2
	Asparagales	Asphodelaceae	*Xanthorrhoea* sp.	ZOTU74	6
	Gentianales	Rubiaceae	UNKNOWN	ZOTU104	5
	Brassicales	Brassicaceae	*Brassica* sp.	ZOTU83	3
			UNKNOWN	ZOTU84	1
	Caryophyllales	Polygonaceae	UNKNOWN	ZOTU88	3
	Fagales	Juglandaceae	UNKNOWN	ZOTU101	3
	Geraniales	Geraniaceae	*Geranium* sp.	ZOTU105	3
	Solanales	Solanaceae	*Solanum* sp.	ZOTU151	3
			*Capsicum* sp.	ZOTU150	2
	Zingiberales	Musaceae	UNKNOWN	ZOTU153	3
	Malpighiales	Euphorbiaceae	*Amperea xiphoclada*	ZOTU109	2
	Arecales	Arecaceae	UNKNOWN	ZOTU70	1
	Fagales	Casuarinaceae	*Casuarina* sp.	ZOTU100	1
	Lamiales	Oleaceae	*Ligustrum* sp.	ZOTU106	1
		Plantaginaceae	*Plantago* sp.	ZOTU107	1
		Scrophulariaceae	*Leucophyllum mojinense*	ZOTU108	1
	Rosales	Rhamnaceae	UNKNOWN	ZOTU143	1
		Rosaceae	*Rosa* sp.	ZOTU144	1
			UNKNOWN	ZOTU145	1
		Ulmaceae	UNKNOWN	ZOTU146	1
	Sapindales	Rutaceae	UNKNOWN	ZOTU148	1
	Vitales	Vitaceae	UNKNOWN	ZOTU152	1
Pinopsida	Pinales	Pinaceae	UNKNOWN	ZOTU157	16
	Cupressales	Cupressaceae	*Callitris* sp.	ZOTU155	5
			*Hesperocyparis* sp.	ZOTU156	4
Polypodiopsida	Polypodiales	Dennstaedtiaceae	UNKNOWN	ZOTU160	16
	Cyatheales	Dicksoniaceae	*Calochlaena* sp.	ZOTU158	2
			*Dicksonia* sp.	ZOTU159	1
Bryopsida	Hypnodendrales	Racopilaceae	*Racopilum* sp.	ZOTU61	2

**TABLE A4:** List of food species detected for bush rat scat samples.

Class	Order	Family	Genus/Species	ZOTU ID	Sum of frequency of occurrence
Bush rat (Rattus *fuscipes*) Scat samples = 49					
*List of arthropods detected from COI metabarcode (Unique ZOTUs = 36)*					
Insecta	Coleoptera	Carabidae	UNKNOWN	ZOTU4	21
		Dermestidae	UNKNOWN	ZOTU6	7
		Cleridae	*Eleale* sp.	ZOTU5	1
	Diptera	Phoridae	UNKNOWN	ZOTU10	11
			UNKNOWN	ZOTU11	4
			*Megaselia* sp.	ZOTU9	2
		Ceratopogonidae	UNKNOWN	ZOTU7	2
		Chironomidae	*Polypedilum* sp.	ZOTU8	1
	Lepidoptera		UNKNOWN	ZOTU57	3
		Hepialidae	*Elhamma australasiae*	ZOTU25	6
			*Oxycanus* sp.	ZOTU26	2
		Limacodidae	*Doratifera oxleyi*	ZOTU28	6
		Zygaenidae	*Myrtartona coronias*	ZOTU55	4
		Oecophoridae	*Heliocausta oecophorella*	ZOTU42	3
			*Ericrypsina* sp.	ZOTU39	1
			*Eulechria suffusa*	ZOTU40	1
		Erebidae	*Philenora omophanes*	ZOTU18	2
			*Praxis aterrima*	ZOTU19	2
		Geometridae	*Idiodes apicata*	ZOTU22	2
			*Chrysolarentia trygodes*	ZOTU21	1
			*Idiodes siculoides*	ZOTU23	1
		Noctuidae	*Thoracolopha spilocrossa*	ZOTU35	2
			*Agrotis porphyricollis*	ZOTU30	1
		Tortricidae	*Bathrotoma constrictana*	ZOTU54	2
		Cosmopterigidae	*Macrobathra chrysotoxa*	ZOTU13	1
		Elachistidae	*Agriophara platyscia*	ZOTU14	1
		Erebidae	*Phaeophlebosia furcifera*	ZOTU17	1
			*Praxis difficilis*	ZOTU20	1
		Lasiocampidae	*Porela albifinis*	ZOTU27	1
		Psychidae	*Clania ignobilis*	ZOTU47	1
			*Lepidoscia retinochra*	ZOTU48	1
		Tineidae	*Opogona stereodyta*	ZOTU52	1
			*Tineola bisselliella*	ZOTU53	1
	Blattodea	Blaberidae	*Calolampra* sp.	ZOTU3	1
	Neuroptera	Osmylidae	*Stenosmylus tenuis*	ZOTU59	1
Malacostraca	Decapoda	Parastacidae	*Geocharax gracilis*	ZOTU60	2
*List of plants detected from trnL metabarcode (Unique ZOTUs = 65)*					
Streptophyta			UNKNOWN	ZOTU162	39
Magnoliopsida			UNKNOWN	ZOTU154	29
	Myrtales	Myrtaceae	UNKNOWN	ZOTU115	38
			UNKNOWN	ZOTU114	37
	Asterales	Asteraceae	UNKNOWN	ZOTU78	30
			*Aster* sp.	ZOTU77	22
		Campanulaceae	*Lobelia anceps*	ZOTU79	7
			UNKNOWN	ZOTU81	6
			*Wahlenbergia marginata*	ZOTU80	3
		Goodeniaceae	*Goodenia rosulata*	ZOTU82	1
	Fabales	Fabaceae	UNKNOWN	ZOTU99	21
			*Acacia* sp.	ZOTU93	17
			*Pultenaea* sp.	ZOTU96	4
			*Viminaria juncea*	ZOTU98	2
			*Templetonia egena*	ZOTU97	1
	Poales	Restionaceae	*Leptocarpus* sp.	ZOTU138	17
			*Hypolaena* sp.	ZOTU137	11
			*Centrolepis monogyna*	ZOTU136	4
			UNKNOWN	ZOTU139	3
		Poaceae	*Microlaena* sp.	ZOTU132	8
			UNKNOWN	ZOTU135	3
			*Cenchrus* sp.	ZOTU129	1
		Cyperaceae	*Gahnia* sp.	ZOTU119	4
			*Lepidosperma tortuosum*	ZOTU120	2
			*Machaerina* sp.	ZOTU123	1
		Juncaceae	*Juncus* sp.	ZOTU126	2
			*Luzula* sp.	ZOTU127	2
	Fagales	Juglandaceae	UNKNOWN	ZOTU101	4
		Casuarinaceae	*Casuarina* sp.	ZOTU100	15
	Proteales	Proteaceae	*Persoonia* sp.	ZOTU141	15
			UNKNOWN	ZOTU142	3
			*Isopogon anemonifolius*	ZOTU140	1
	Asparagales	Asphodelaceae	*Xanthorrhoea* sp.	ZOTU74	10
		Iridaceae	UNKNOWN	ZOTU75	10
		Orchidaceae	UNKNOWN	ZOTU76	4
		Asparagaceae	*Lomandra multiflora*	ZOTU73	3
	Gentianales	Rubiaceae	UNKNOWN	ZOTU104	10
		Loganiaceae	*Mitrasacme pilosa*	ZOTU102	6
	Apiales		UNKNOWN	ZOTU69	7
		Araliaceae	*Hydrocotyle* sp.	ZOTU66	7
			*Panax* sp.	ZOTU68	2
			*Macropanax dispermus*	ZOTU67	1
		Apiaceae	*Centella* sp.	ZOTU63	3
			UNKNOWN	ZOTU65	2
			*Mackinlaya* sp.	ZOTU64	1
	Sapindales	Rutaceae	*Boronia* sp.	ZOTU148	5
			UNKNOWN	ZOTU149	1
	Dilleniales	Dilleniaceae	*Tetracera* sp.	ZOTU91	4
			*Tetracera nordtiana*	ZOTU90	3
	Malpighiales	Violaceae	*Melicytus dentatus*	ZOTU111	3
	Rosales	Rosaceae	*Rosa* sp.	ZOTU144	2
	Solanales	Solanaceae	*Solanum* sp.	ZOTU151	2
	Zingiberales	Musaceae	UNKNOWN	ZOTU153	2
	Brassicales	Brassicaceae	*Brassica* sp.	ZOTU83	1
	Caryophyllales	Droseraceae	*Drosera erythrorhiza*	ZOTU86	1
	Celastrales	Celastraceae	*Stackhousia* sp.	ZOTU89	1
	Ericales	Theaceae	UNKNOWN	ZOTU92	1
	Lamiales	Plantaginaceae	*Plantago* sp.	ZOTU107	1
	Malpighiales	Euphorbiaceae	*Amperea xiphoclada*	ZOTU109	1
	Malvales	Thymelaeaceae	*Pimelea* sp.	ZOTU113	1
	Oxalidales	Elaeocarpaceae	*Tetratheca* sp.	ZOTU117	1
	Santalales	Loranthaceae	*Muellerina eucalyptoides*	ZOTU147	1
Polypodiopsida	Polypodiales	Dennstaedtiaceae	UNKNOWN	ZOTU160	9
Pinopsida	Pinales	Pinaceae	UNKNOWN	ZOTU157	4
Lycopodiopsida	Isoetales	Isoetaceae	*Isoetes* sp.	ZOTU62	2

**TABLE A5:** List of food species detected for heath mouse scat samples.

Class	Order	Family	Genus/Species	ZOTU ID	Sum of frequency of occurrence
Heath mouse (*Pseudomys shortridgei*) Scat samples = 31					
*List of arthropods detected from COI metabarcode (Unique ZOTUs = 15)*					
Insecta	Coleoptera	Carabidae	UNKNOWN	ZOTU4	4
		Dermestidae	UNKNOWN	ZOTU6	2
	Diptera	Phoridae	UNKNOWN	ZOTU10	3
	Lepidoptera		UNKNOWN	ZOTU57	2
		Psychidae	*Clania ignobilis*	ZOTU47	2
		Erebidae	*Phaeophlebosia furcifera*	ZOTU17	1
			*Praxis aterrima*	ZOTU19	1
		Limacodidae	*Doratifera oxleyi*	ZOTU28	1
		Oecophoridae	*Garrha* sp.	ZOTU41	1
			UNKNOWN	ZOTU45	1
		Pyralidae	*Plodia interpunctella*	ZOTU50	1
		Tineidae	*Tineola bisselliella*	ZOTU53	1
		Zygaenidae	*Myrtartona coronias*	ZOTU55	1
			*Pollanisus viridipulverulenta*	ZOTU56	1
Arachnida	Araneae	Araneidae	*Argiope protensa*	ZOTU1	1
*List of plants detected from trnL metabarcode (Unique ZOTUs = 62)*					
Streptophyta			UNKNOWN	ZOTU162	27
Magnoliopsida			UNKNOWN	ZOTU154	5
	Poales	Restionaceae	*Hypolaena* sp.	ZOTU137	23
			*Leptocarpus* sp.	ZOTU138	23
			*Centrolepis monogyna*	ZOTU136	1
			UNKNOWN	ZOTU139	1
		Cyperaceae	*Schoenus apogon*	ZOTU124	6
			*Lepidosperma* sp.	ZOTU121	3
			*Lepidosperma tortuosum*	ZOTU120	3
			*Machaerina gunnii*	ZOTU122	2
			*Machaerina* sp.	ZOTU123	1
			*Schoenus lepidosperma*	ZOTU125	1
		Poaceae	*Stipa* sp.	ZOTU134	5
			*Microlaena* sp.	ZOTU132	3
			UNKNOWN	ZOTU135	2
			*Holcus* sp.	ZOTU130	1
	Gentianales	Rubiaceae	UNKNOWN	ZOTU104	20
		Rubiaceae	*Lasianthus* sp.	ZOTU103	1
		Loganiaceae	*Mitrasacme pilosa*	ZOTU102	4
	Myrtales		UNKNOWN	ZOTU115	17
		Myrtaceae	UNKNOWN	ZOTU114	19
		Fabaceae	UNKNOWN	ZOTU99	16
			*Acacia* sp.	ZOTU93	4
			*Daviesia* sp.	ZOTU94	2
			*Pultenaea* sp.	ZOTU96	1
	Fagales	Casuarinaceae	*Casuarina* sp.	ZOTU100	13
		Juglandaceae	UNKNOWN	ZOTU101	3
	Asterales	Asteraceae	UNKNOWN	ZOTU78	12
			*Aster* sp.	ZOTU77	9
		Campanulaceae	*Wahlenbergia marginata*	ZOTU80	2
			UNKNOWN	ZOTU81	2
		Goodeniaceae	*Goodenia rosulata*	ZOTU82	1
	Proteales	Proteaceae	*Isopogon anemonifolius*	ZOTU140	10
			*Persoonia* sp.	ZOTU141	9
			UNKNOWN	ZOTU142	4
	Asparagales	Asphodelaceae	*Xanthorrhoea* sp.	ZOTU74	9
		Asparagaceae	*Lomandra multiflora*	ZOTU73	4
		Iridaceae	UNKNOWN	ZOTU75	4
		Amaryllidaceae	*Allium* sp.	ZOTU72	1
		Orchidaceae	UNKNOWN	ZOTU76	1
	Sapindales	Rutaceae	*Boronia* sp.	ZOTU148	7
			UNKNOWN	ZOTU149	2
	Dilleniales	Dilleniaceae	*Tetracera nordtiana*	ZOTU90	4
			*Tetracera* sp.	ZOTU91	4
	Apiales		UNKNOWN	ZOTU69	3
		Apiaceae	UNKNOWN	ZOTU65	1
		Araliaceae	*Panax* sp.	ZOTU68	1
	Malvales	Thymelaeaceae	*Pimelea* sp.	ZOTU113	2
	Solanales	Solanaceae	*Capsicum* sp.	ZOTU150	2
	Pinales	Pinaceae	UNKNOWN	ZOTU157	2
	Isoetales	Isoetaceae	*Isoetes* sp.	ZOTU62	1
	Caryophyllales	Droseraceae	*Drosera erythrorhiza*	ZOTU86	1
		Polygonaceae	*Persicaria* sp.	ZOTU87	1
	Lamiales	Plantaginaceae	*Plantago* sp.	ZOTU107	1
	Malpighiales	Euphorbiaceae	*Amperea xiphoclada*	ZOTU109	1
	Oxalidales	Elaeocarpaceae	*Tetratheca* sp.	ZOTU117	1
	Rosales	Rosaceae	*Rosa* sp.	ZOTU144	1
		Ulmaceae	UNKNOWN	ZOTU146	1
	Santalales	Loranthaceae	*Muellerina eucalyptoides*	ZOTU147	1
	Zingiberales	Musaceae	UNKNOWN	ZOTU153	1
Pinopsida	Cupressales	Cupressaceae	*Hesperocyparis* sp.	ZOTU156	1
Polypodiopsida	Schizaeales	Schizaeaceae	*Schizaea elegans*	ZOTU161	1
